# Pretreatment Inflammatory Indexes as Prognostic Predictors of Survival in Patients Suffering From Synovial Sarcoma

**DOI:** 10.3389/fonc.2019.00955

**Published:** 2019-09-24

**Authors:** Yuan Cheng, Fei Mo, Lutong Pu, Qingfang Li, Xuelei Ma

**Affiliations:** State Key Laboratory of Biotherapy and Cancer Center, West China Hospital, Sichuan University and Collaborative Innovation Center, Chengdu, China

**Keywords:** synovial sarcoma, inflammatory biomarkers, neutrophil-to-lymphocyte ratio (NLR), platelet-to-lymphocyte ratio (PLR), lymphocyte-to-monocyte ratio (LMR), survival

## Abstract

**Background:** Inflammatory indexes have been considered as important prognostic factors in various types of cancers. This study aimed to evaluate prognostic values of neutrophil-to-lymphocyte ratio (NLR), platelet-to-lymphocyte ratio (PLR), lymphocyte-to-monocyte ratio (LMR) in patients with synovial sarcoma (SS).

**Methods:** One hundred and three patients diagnosed with SS were collected during 2006–2017 and divided into high or low NLR, PLR, and LMR groups based on receiver operating characteristic curve analysis. Data of clinical variables were collected for univariate and multivariate analyses. The Kaplan–Meier method was used to analyze OS and PFS of SS patients and significance was evaluated by the log-rank test.

**Results:** The optimal cut-off values of NLR, PLR, and LMR were 2.70, 154.99, and 4.16, respectively. Univariate analyses identified resection surgery, distant metastasis, NLR, PLR, and LMR as the potential predictors of progression-free survival (PFS) and overall survival (OS). In the multivariate analyses, NLR was independent predictors for OS (HR 5.074, 95% CI 1.200–21.463, *p* = 0.027). Resection surgery, metastasis and LMR was independent predictors for PFS (HR 5.328, *p* = 0.017; HR 3.114, *p* = 0.04 and HR 0.202, *p* = 0.025, respectively).

**Conclusion:** Resection surgery, distant metastasis, NLR, and LMR were independent prognostic factors of PFS and OS in patients with synovial sarcoma. Surgery as an effective treatment strategy, other than radiotherapy and chemotherapy, can significantly prolong survival of synovial patients. Clinical utility of these inflammatory biomarkers should be validated in a larger sample size study.

## Introduction

Soft tissue sarcomas (STSs) are mesenchymal malignant tumors, accounting for <1% of all malignant tumors and 2% of all cancer-related deaths (1). Although synovial sarcoma (SS) accounts for only ~5 to 10% of all STSs, it is the commonest non-rhabdomyosarcomatous soft tissue sarcoma in adolescent and young adults (1, 2). SS was once thought to originate from synovial cells due to its frequent occurrence in soft tissue around joint. However, it has been found in almost every part of the body with a rare frequency and the specific cellular origin remains unclear (3, 4). SS is generally considered as a high-grade sarcoma, with 5-, 10-, and 15-year survival rates survival rates of ~60, 50, and 45%, respectively (5). SS tends to occur in young people, with a slight male predominance, and mostly affects extremities (>80%) (6). The tumors can be divided into three histological types: biphasic (consist of both spindle and epithelioid cells), monophasic (only spindle cells component) and poorly differentiated (containing small round cells). Despite the morphological difference, they are histogenetically similar through the presence of the t(X;18)(p11.2;q11.2) translocation (7, 8).

It is known that tumor size (<5 cm), age of patients (<20 years old), radiotherapy and complete resection surgery are important positive prognostic factors for patients with SS (5, 9). Whereas, smaller SSs unexpectedly have a poor prognosis during occasional cases (9). In another cohort, age <35 years is a main predictor for patients' prognosis (6). Treatment strategies for SS involve surgery, chemotherapy and radiotherapy. Surgery is an optimal choice for localized tumor, which is usually combined with radiotherapy. Radiotherapy aims to decrease tumor size and help in delaying local invasion. Patients with standard care of surgery and radiotherapy usually have a good chance to control localized disease (10). Although SS is considered to be sensitive to chemotherapy, especially to alkylating agents like ifosfamide and doxorubicin, when compared with other adult soft tissue sarcoma, the response rate still remains about 50% (11, 12). Whereas, routine administration of chemotherapy is of no benefit in reducing systemic relapse in pediatric patients (10). The therapeutic effects of both chemotherapy and radiotherapy vary from different types and stages of SS (13). Therefore, identifying high-risk SS through a different way might be helpful in management of this disease.

Recently, increasing evidence has revealed that systemic inflammatory response plays a remarkable role in prognosis of various malignant tumors, including colorectal cancer, breast cancer, gastric cancer, esophageal cancer, ovarian cancer and pancreatic cancer (14–19). For soft tissue sarcoma, previous investigates have also indicated that inflammatory indexes, such as neutrophil-to-lymphocyte ratio (NLR), platelet-to-lymphocyte ratio (PLR), lymphocyte-to-monocyte ratio (LMR) and absolute lymphocyte count (ALC), are independent prognostic biomarkers for osteosarcoma, Ewing sarcoma and rhabdomyosarcoma (20–22). Recent years, several studies have focused on prognostic factors for synovial sarcoma. High NLR was found to be a reliable prognostic factor which was associated with worse survival for synovial sarcoma patients (23, 24).

The aim of this study was to estimate the prognostic values of not only pre-treatment NLR, but also PLR and LMR in SS patients and identify high-risk patients for better management.

## Patients and Methods

### Patients

The Medical Ethics Committee of West China Hospital approved this study before this study launch. We retrospectively reviewed the medical records of all newly diagnosed synovial sarcoma patients between January, 2005 and December, 2017 in West China Hospital. The inclusion criteria were as follows: (a) patient with SS confirmed by histopathology; (b) patients without previous anti-cancer treatment, including surgery resection, chemotherapy and radiotherapy; (c) patients with informed consent. The exclusion criteria included: (a) patients with obvious infection or autoimmune diseases; (b) patients with hematologic diseases; (d) patients suffered from other malignant diseases; (e) patients without sufficient data for further analysis. Finally, 103 patients were included in this study. Each patient was followed up regularly until death or December 2017. The follow-up interval varied from 6 month to 1 year.

### Data Extraction and Inflammatory Indexes Analysis

Clinical features, including age, sex, tumor location, metastasis at diagnosis, tumor size, treatment strategy, and laboratory index values, such as neutrophil counts, lymphocyte counts, platelet counts, monocyte counts, LDH, were extracted from the medical records of the enrolled patients. OS was measured as the period between the date of diagnosis of SS and the date of death. PFS was calculated from the date of diagnosis to the date of disease relapse and progression. The date of last follow-up was used for drop-out patients. NLR and PLR were defined as the ratio of absolute neutrophil counts and platelet counts divided by the absolute lymphocyte counts, respectively. LMR was defined as the absolute lymphocyte counts divided by the absolute monocyte counts. Patients with complete resection surgery were refer to those who have undertaken surgery treatment, whereas patients with margin status R1 or R2 were refer to no surgery treatment.

### Statistical Analysis

Receiver operating characteristic (ROC) curve was applied to evaluate the sensitivity of the inflammatory indexes and Youden index was identified as the optimal cut-off value. Student's *t*-test was used to exam the difference of continuous variables. Comparison of categorical variables, Chi-square test or the Fisher exact test was applied. Survival curves were plotted by Kaplan–Meier analysis and Log-rank test was performed to identify the significance of the difference. Significant variables for OS or PFS were identified by univariate analysis and then further evaluated by multivariate analysis using Cox's proportional hazard regression analysis. *P*-values were based on two-tail test and <0.05 were considered statistically significant. All statistical analysis was performed by using SPSS version 19.0 (IBM Corporation, Armonk, NY, USA).

## Results

### Baseline Characteristics

A total of 149 patients with synovial sarcoma were identified from our database and 103 patients were finally enrolled. The cut-off values of NLR, PLR, and LMR were 2.70, 154.99, and 4.16, respectively. The baseline characteristics of patients are shown in Table 1.

**Table 1:** Baseline characteristics of the patients with synovial sarcoma.

**Clinical parameters**	**Total**	**NLR**		***p*-value**	**PLR**		***p*-value**	**LMR**		***p*-value**
	***N* = 103**	** <2.70**	**≥2.70**		** <154.99**	**≥154.99**		**≥4.16**	** <4.16**	
	**n (%)**	**n (%)**	**n (%)**		**n (%)**	**n (%)**		**n (%)**	**n (%)**	
Median age, years (range)	37 (1–78)	35 (17–78)	37 (1–74)	0.890	37 (1–74)	33 (13–78)	0.280	36 (1–70)	37 (17–78)	0.636
**Gender**										
Female	49 (47.6)	39 (53.4)	10 (33.3)	0.083	39 (52.0)	10 (35.7)	0.184	37 (54.4)	12 (34.3)	0.063
Male	54 (52.4)	34 (46.6)	20 (66.7)		36 (48.0)	18 (64.3)		31 (45.6)	23 (65.7)	
**Surgery**										
No	8 (7.8)	5 (6.8)	3 (10.0)	0.689	5 (6.7)	3 (10.7)	0.680	5 (7.4)	3 (8.6)	1.000
Yes	95 (92.2)	68 (93.2)	27 (90.0)		70 (93.3)	25 (89.3)		63 (92.6)	32 (91.4)	
**Radiotherapy**										
No	84 (81.6)	60 (82.2)	24 (80.0)	0.785	63 (84.0)	21 (75.0)	0.391	57 (83.8)	27 (77.1)	0.430
Yes	19 (18.4)	13 (17.8)	6 (20.0)		12 (16.0)	7 (25.0)		11 (16.2)	8 (22.9)	
**Chemotherapy**										
No	71 (68.9)	56 (76.7)	15 (50.0)	0.010	57 (76.0)	14 (50.0)	0.016	51 (75.0)	20 (57.1)	0.075
Yes	32 (31.1)	17 (23.3)	15 (50.0)		18 (24.0)	14 (50.0)		17 (25.0)	15 (42.9)	
**Relapse**										
No	62 (60.2)	44 (60.30)	18 (60.0)	1.000	46 (61.3)	16 (57.1)	0.822	40 (58.8)	22 (62.9)	0.832
Yes	41 (39.8)	29 (39.7)	12 (40.0)		29 (38.7)	12 (42.9)		28 (41.20	13 (37.1)	
**Metastasis**										
No	81 (78.6)	63 (86.3)	18 (60.0)	0.007	65 (86.7)	16 (57.1)	0.002	58 (85.3)	23 (65.7)	0.040
Yes	22 (21.4)	10 (13.7)	12 (40.0)		10 (13.3)	12 (42.9)		10 (14.7)	12 (34.3)	
**Tumor location**										
Internal organs	18 (17.5)	10 (13.7)	8 (26.7)	0.062	9 (12.0)	9 (32.1)	0.011	6 (8.8)	12 (34.3)	0.005
Extremities	56 (54.4)	45 (61.6)	11 (36.7)		47 (62.70	9 (32.1)		42 (61.8)	14 (40.0)	
Trunk	29 (28.2)	18 (24.7)	11 (36.7)		19 (25.3)	10 (35.7)		20 (29.4)	9 (25.7)	
**T stage^†^**										
<5 cm	24 (23.3)	20 (31.3)	4 (16.7)	0.193	20 (30.30	4 (18.2)	0.408	18 (30.5)	6 (20.7)	0.447
≥5 cm	64 (62.1)	44 (68.8)	20 (83.3)		46 (69.7)	18 (81.8)		41 (69.5)	23 (79.3)	
**LDH**										
<169.5	52 (50.5)	39 (53.4)	13 (43.3)	0.391	42 (56.0)	10 (35.7)	0.079	38 (55.9)	14 (40.0)	0.149
≥169.5	51 (49.5)	34 (46.6)	17 (56.7)		33 (44.0)	18 (64.3)		30 (44.1)	21 (60.0)	

† *88 were available. NLR neutrophil-lymphocyte ratio, PLR platelet-lymphocyte ratio, LMR lymphocyte-monocyte ratio, LDH lactate dehydrogenase*.

SS tended to occur in younger people, with median age of patients was 37 (range 1–78) years. There were 54 (52.4%) males and 49 (47.6%) females. Most patients (95, 92.2%) received surgery, whereas only 19 (18.4%) and 32 (31.1%) received radiotherapy and chemotherapy, respectively. During the follow-up period, 41 (39.8%) patients experienced disease relapse and 22 (21.4%) patients had distant metastasis. Of note, metastasis was significantly associated with NLR, PLR and LMR. Patients with high NLR, PLR and lower LMR were likely to develop distant metastasis. Pathological results suggested extremities were the most common sites for SS (56, 54.4%). Eighteen (17.5%) and 29 (28.2%) patients had tumor located in internal organ and trunk, respectively. Of the entire patients, 64 (62.1%) patients had tumors larger than 5 cm.

The median overall survival (OS) and median progression-free survival (PFS) was 44.0 months (95% confidence interval [CI] 37.0–57.0) and 25.0 months (95% CI 14.6–36.0), respectively. We explored associations of NLR, PLR, and LMR with these baseline characteristics and results suggested chemotherapy, distant metastasis and tumor location were statistically significantly associated with NLR, PLR, or LMR (*p* < 0.05).

### Univariate Analyses and Multivariate Analyses

We investigated the associations between patients' baseline characteristics, including NLR, PLR, and LMR, and survival by using Cox's proportional hazard regression analysis. Univariate analyses indicated that resection surgery, distant metastasis, tumor location, NLR, PLR, and LMR were closely correlated with prognosis of patients (*p* < 0.1). Furthermore, multivariate analyses of OS and PFS were performed including markers mentioned above to identify independent predictor for survival (Tables 2, 3).

**Table 2:** Summary of univariate and multivariate analysis for OS in patients with synovial sarcoma.

**Parameter**	**Average OS**	**95% CI**	**Univariate analysis**		**Multivariate analysis**	
			**HR (95% CI)**	***p*-value**	**HR (95% CI)**	***p*-value**
**Gender**						
Male	75.6	67.6–83.6	1.000	0.628	–	–
Female	89.4	80.1–98.6	0.782 (0.289–2.118)		–	–
**Age**						
≥37	86.0	76.0–96.1	1.000	0.542	–	–
<37	82.0	74.4–89.5	0.735 (0.274–1.976)		–	–
**Surgery**						
No	58.7	28.9–88.6	1.000	0.058	1.000	0.103
Yes	89.6	82.9–96.3	0.295 (0.084–1.041)		3.210 (0.791–13.027)	
**Radiotherapy**						
No	88.1	80.8–95.3	1.000	0.610	–	–
Yes	84.5	68.8–100.3	1.342 (0.433–4.162)		–	–
**Chemotherapy**						
No	89.9	82.6–97.3	1.000	0.322	–	–
Yes	82.6	69.7–95.5	1.648 (0.613–4.426)		–	–
**Relapse**						
No	90.2	82.1–98.4	1.000	0.243	–	–
Yes	83.3	72.0–94.7	1.804 (0.670–4.856)		–	–
**Metastasis**						
No	93.3	87.0–99.7	1.000	0.009	1.000	0.165
Yes	70.6	53.6–87.6	3.713 (1.392–9.908)		2.331 (0.706–7.699)	
**Tumor location**						
Internal organs	58.5	41.5–75.4	1.000	0.062	1.000	
Extremities	89.9	81.6–98.2	0.326 (0.109–0.977)	0.045	0.391 (0.097–1.582)	0.188
Trunk	93.2	82.9–103.6	0.241 (0.059–0.980)	0.047	0.258 (0.056–1.183)	0.081
**T stage**						
<5 cm	85.8	77.5–94.1	1.000	0.232	–	–
≥5 cm	77.3	69.4–85.3	2.493 (0.557–11.152)		–	–
**LDH**						
<169.5	88.4	79.2–97.7	1.000	0.924	–	–
≥169.5	79.0	70.8–87.2	1.049 (0.393–2.798)		–	–
**NLR**						
<2.70	86.0	80.7–91.3	1.000	0.003	1.000	**0.027**
≥2.70	71.0	55.1–86.9	4.651 (1.688–12.811)		5.074 (1.200–21.463)	
**PLR**						
<154.99	84.0	78.1–90.0	1.000	0.037	1.000	0.167
≥154.99	76.0	60.4–91.5	2.832 (1.062–7.553)		3.195 (0.615–16.589)	
**LMR**						
<4.16	71.2	56.5–85.9	1.000	0.002	1.000	0.056
≥4.16	96.1	90.3–101.9	0.190 (0.066–0.547)		0.280 (0.076–1.035)	

*OS overall survival, LDH lactate dehydrogenase, NLR neutrophil-lymphocyte ratio, PLR platelet-lymphocyte ratio, LMR lymphocyte-monocyte ratio*.

**Table 3:** Summary of univariate and multivariate analysis for PFS in patients with synovial sarcoma.

**Parameter**	**Average PFS**	**95% CI**	**Univariate analysis**		**Multivariate analysis**	
			**HR (95% CI)**	***p*-value**	**HR (95% CI)**	***p*-value**
**Gender**						
Male	73.9	54.9–82.9	1.000	0.809	–	–
Female	85.3	73.6–97.0	1.130 (0.420–3.038)		–	–
**Age**						
≥37	82.8	71.4–94.3	1.000	0.619	–	–
<37	74.0	65.8–82.3	0.778 (0.290–2.090)		–	–
**Surgery**						
No	P	20.8–68.2	1.000	0.056	1.000	**0.017**
Yes	87.2	79.7–94.7	0.292 (0.083–1.031)		5.328 (1.349–21.041)	
**Radiotherapy**						
No	86.8	78.9–94.8	1.000	0.643	–	–
Yes	73.3	58.0–88.7	1.309 (0.420–4.074)		–	–
**Chemotherapy**						
No	88.2	79.7–96.6	1.000	0.269	–	–
Yes	69.5	56.7–82.2	1.748 (0.650–4.698)		–	–
**Relapse**						
No	90.5	82.4–98.5	1.000	0.074	1.000	**0.034**
Yes	48.1	35.8–60.4	2.511 (0.915–6.892)		3.301 (1.094–9.964)	
**Metastasis**						
No	81.5	75.2–87.8	1.000	0.004	1.000	**0.040**
Yes	63.5	42.5–84.6	4.186 (1.568–11.179)		3.114 (1.054–9.199)	
**Tumor location**						
Internal organs	38.2	24.5–52.0	1.000	0.227	–	–
Extremities	88.3	78.8–97.8	0.360 (0.105–1.235)	0.104	–	–
Trunk	76.3	66.0–86.6	0.384 (0.103–1.430)	0.154	–	–
**T stage**						
<5 cm	82.7	71.7–93.7	1.000	0.240	–	–
≥5 cm	71.6	62.8–80.5	2.456 (0.549–10.983)		–	–
**LDH**						
<169.5	85.7	75.1–96.0	1.000	0.870	–	–
≥169.5	76.2	66.7–85.6	1.085 (0.407–2.893)		–	–
**NLR**						
<2.70	82.7	76.3–89.1	1.000	0.003	1.000	0.098
≥2.70	67.0	49.3–84.6	4.653 (1.686–12.847)		3.361 (0.801–14.102)	
**PLR**						
<154.99	80.4	73.5–87.4	1.000	0.027	1.000	0.226
≥154.99	72.2	54.7–89.6	3.040 (1.137–8.125)		2.671 (0.544–13.116)	
**LMR**						
<4.16	69.0	52.8–85.1	1.000	0.003	1.000	**0.025**
≥4.16	83.7	77.5–89.8	0.199 (0.069–0.574)		0.202 (0.050–0.821)	

*PFS progression-free survival, LDH lactate dehydrogenase, NLR neutrophil-lymphocyte ratio, PLR platelet-lymphocyte ratio, LMR lymphocyte-monocyte ratio*.

Treatment strategies for SS remained unclear, however, our results suggested patients received resection surgery had better PFS (87.2 vs. 44.5 months, *p* = 0.056) and OS (89.6 vs. 58.7 months, *p* = 0.058) than those without resection surgery. Resection surgery was shown to be an independent indicator for PFS (hazard ratio [HR] 5.328, 95% CI 1.349–21.041, *p* = 0.017), not for OS. Patients without distant metastasis shared favorable PFS (81.5 months vs. 63.5 months, *p* < 0.01 and OS (93.3 months vs. 70.6 months, *p* < 0.01). Metastasis was independent indicator for PFS (HR 3.114, 95% CI 1.054–9.199, *p* = 0.04). SS that initially occurred in internal organs, such as lung, kidney and mediastinum revealed poor outcomes, with shorter OS and PFS (compared to extremities, 58.5 vs. 89.9 months, *p* = 0.045 and 38.2 vs. 88.3 months, *p* = 0.104). Compared to patients with higher NLR and PLR, patients in lower NLR and PLR groups were shown to have better PFS (82.7 months vs. 67.0 months, *p* < 0.01 and 80.4 months vs. 72.2 months, *p* < 0.05, respectively) and OS (86.0 months vs. 71.0 months, *p* < 0.01 and 84.0 months vs. 76.0 months, *p* < 0.05, respectively). On the contrary lower LMR was a marker for shorter PFS (69.0 vs. 83.7 months, *p* < 0.01) and OS (71.2 vs. 96.1 months, *p* < 0.01). NLR was an independent predictor for OS, with higher NLR associated with poor prognosis (HR 5.074, 95% CI 1.200–21.463, *p* = 0.027). Higher LMR, as an independent indicator for PFS, was significantly associated with better PFS (HR 0.202, 95% CI 0.050–0.821, *p* = 0.025). However, PLR was not independent indicator for either OS or PFS. Other characteristics, including gender, age, radiotherapy, chemotherapy, tumor size and LDH, were not shown to be associated with PFS and OS.

### Kaplan-Meier Survival Analysis

**Figure 1:**
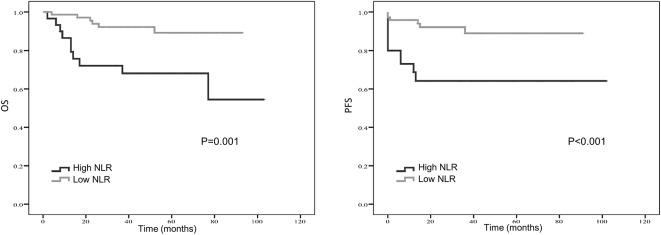
Kaplan-Meier estimates of overall survival (OS) and progression-free survival (PFS) probability according to pre-treatment neutrophil-to-lymphocyte ratio (NLR) level.

**Figure 2:**
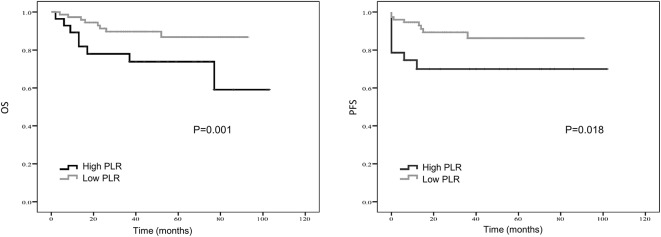
Kaplan-Meier estimates of overall survival (OS) and progression-free survival (PFS) probability according to pre-treatment platelet-to- lymphocyte ratio (PLR) level.

**Figure 3:**
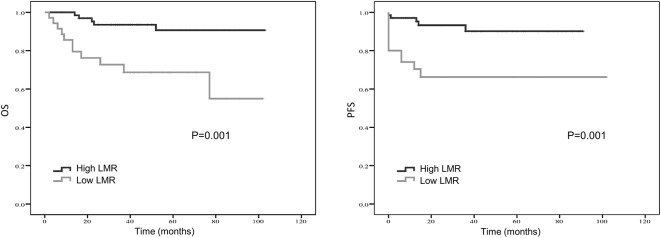
Kaplan-Meier estimates of overall survival (OS) and progression-free survival (PFS) probability according to pre-treatment lymphocyte-to-monocyte-ratio (LMR) level.

**Figure 4:**
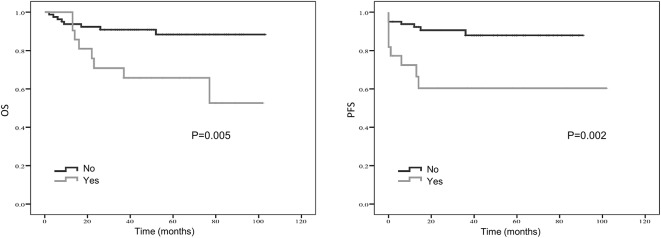
Kaplan-Meier estimates of overall survival (OS) and progression-free survival (PFS) probability according to localized vs. metastatic disease.

**Figure 5:**
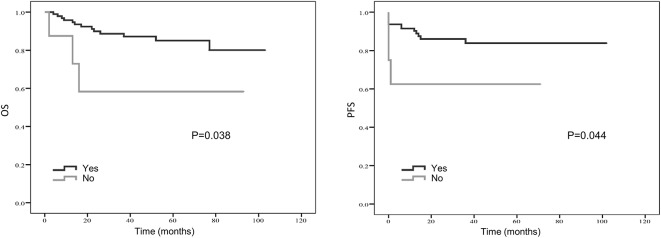
Kaplan-Meier estimates of overall survival (OS) and progression-free survival (PFS) probability according to whether surgery was applied.

Kaplan–Meier curve showed that distant metastasis, NLR, PLR, LMR, and surgery were significantly associated with PFS and OS (Figures 1–5).

## Discussion

Inflammatory indexes as prognostic factors for STSs have recently received more and more attention. Not only individual inflammatory markers, such as CRP, and lymphocytes, but also combination of them, such as NLR, PLR and LMR, have been investigated in STSs (20, 22, 25, 26). NLR was found to be a prognostic inflammatory index for synovial sarcoma (SS) (23, 24). In addition, the overall survival of SS still remains unsatisfying (27). Therefore, the aim of this study was to identify more valuable prognostic indexes for SS and to select patients who were at high risk and required more aggressive treatment strategies. Our results suggested that not undergoing complete resection surgery, distant metastasis, high NLR group, high PLR group, and low LMR group were significantly associated with poor prognosis. Whereas, gender, age, radiotherapy, chemotherapy, tumor size, and LDH were not significantly associated with patients' OS and PFS. Our data failed to demonstrate the prognostic values of tumor size, radiotherapy and chemotherapy. This finds were not only limited by small sample size and non-randomized cohorts, but also because of raising debates of administration of chemotherapy (28–30). Previous studies have proved that high NLR, PLR and low LMR are associated poor prognosis in various malignancies (31–35). Current research also confirmed the prognostic values of these three inflammatory indexes in SS.

NLR, PLR, and LMR are derived from the absolute counts of neutrophils, lymphocytes and monocytes, therefore, the ratios of these three groups of cells in tumor microenvironment play a vital role in predicting the prognosis of patients. Myeloid-derived cells, such as neutrophils and monocytes, are the most abundant hematopoietic cells in human body but usually regarded as potent immune suppressors in tumor microenvironment (36). Myeloid-derived suppressor cells (MDSCs) have recently been widely investigated. This group of cells produces a proinflammatory response and promotes angiogenesis and metastasis of tumor (37–39). Derived from circulating monocytes, tumor-associated macrophages have also been proved to be related to tumor cells proliferation, invasion and metastasis (40, 41). In contrast, tumor-infiltrating lymphocytes are considered important in anti-cancer immune response via producing cytokines and inducing cytotoxic cell death (42). Therefore, lymphocytes are thought to be a positive predictor (43, 44). Individual absolute counts of neutrophils and monocytes are suggested to be independent prognosis factors in various cancers (45–47). Meanwhile, it is known that the immune suppressive effect of MDSCs is mainly based on suppressing the activity of T lymphocytes (36). Productions released by MDSCs, such as Arg1 and iNOS, can block T cells and lead to tumor progression and metastasis (48–50). Increased neutrophils, monocytes and decreased lymphocytes are associated with immune suppressive status, therefore, high NLR and low LMR are associated with poor survival outcomes.

Platelets also interact with tumor cells and decreased platelets level is associated with decreased tumor metastasis (51). Tumor cells can gather platelets and protect themselves from cytolysis of NK cells in human blood. This process promotes migration of tumor cells and tumor metastasis (52). Meanwhile, platelets provide a procoagulant surface to help cancer cells escape from immune response, thus promote cancer growth and dissemination (53). Platelets can also activate several signaling pathways within cancer cells, resulting in transition toward a more invasive mesenchymal-like phenotype (54). In accordance with the critical role of lymphocytes in the suppression of tumor progression, high PLR suggests a rather poor prognosis for cancer patients. Although PLR is significantly associated with poor prognosis in univariate analysis, it was not an independent prognostic factor for OS or PFS, which was consistent with previous studies on soft tissue sarcomas and other malignancies (22, 55). One possible reason is that the immune-suppression and tumor-promotion effect of MDSCs are more sustainable and potent than platelets (56), which makes platelet plays a rather small role.

This study investigated the impact of inflammatory indexes on synovial sarcoma and provided an alternative predictive model for prognosis of SS. However, this research still has several limitations. Firstly, due to the rarity of SS and single-center study, the number of patients (*n* = 103) is limited, which may cause selection bias. The subgroup analyses are also limited by the small sample size. Whereas, SS may behavior differently according to age of presence or histological subtypes. Inflammatory indexes are likely to play different roles in the subgroup analyses. Secondly, we just collected relatively a few clinical predictors and some important indicators may be ignored. Therefore, a larger sample size study with more clinical indicators is required to validate our findings. For more precise evaluation, randomized clinical trials are required.

## Conclusion

In summary, high NLR, high PLR, low LMR, metastasis at diagnosis and no surgery were remarkable risk factors for SS patients. Furthermore, NLR, LMR and metastasis were independent factors for OS and PFS, except for PLR. As a result, NLR and LMR, as inflammatory indexes, were superior to PLR. Surgery could significantly prolong PFS of SS patients. These prognostic indexes might be helpful in making treatment decisions for SS patients with different risks.

## Data Availability Statement

All datasets generated for this study are included in the manuscript/supplementary files.

## Ethics Statement

Approval for this retrospective research was obtained from the institutional review board of West China hospital, Sichuan University. Written informed consent was obtained from all included patients.

## Author Contributions

XM: study concept and design. YC, FM, and QL: acquisition of data. YC and XM: analysis and interpretation of data. YC: drafting of the manuscript. LP and XM: critical revision of the manuscript for important intellectual content. YC, FM, and LP: statistical analysis. XM: study supervision. All authors read and approved the final manuscript.

### Conflict of Interest

The authors declare that the research was conducted in the absence of any commercial or financial relationships that could be construed as a potential conflict of interest.

## References

[1] Weiss SW Goldblum JR Malignant Soft Tissue Tumors Of Uncertain Type (2001).

[2] Speth BM Krieg AH Kaelin A Exner GU Guillou L von HA. Synovial sarcoma in patients under 20 years of age: a multicenter study with a minimum follow-up of 10 years. J Child Orthop. (2011) 5:335–42. 10.1007/s11832-011-0360-4 23024724 PMC3179534

[3] McKinney CD Mills SE Fechner RE. Intraarticular synovial sarcoma. Am J Surg Pathol. (1992) 16:1017–20. 10.1097/00000478-199210000-00014 1384367

[4] Ishida T Iijima T Moriyama S Nakamura C Kitagawa T Machinami R. Intra-articular calcifying synovial sarcoma mimicking synovial chondromatosis. Skeletal Radiol. (1996) 25:766–9. 10.1007/s002560050176 8958625

[5] Bergh P Meis-Kindblom JM Gherlinzoni F Berlin O Bacchini P Bertoni F. Synovial sarcoma: identification of low and high risk groups. Cancer. (1999) 85:2596–607. 10375108 10.1002/(sici)1097-0142(19990615)85:12<2596::aid-cncr16>3.0.co;2-k

[6] Spurrell EL Fisher C Thomas JM Judson IR. Prognostic factors in advanced synovial sarcoma: an analysis of 104 patients treated at the Royal Marsden Hospital. Ann Oncol. (2005) 16:437–44. 10.1093/annonc/mdi082 15653701

[7] Thway K Fisher C. Synovial sarcoma: defining features and diagnostic evolution. Ann Diagn Pathol. (2014) 18:369–80. 10.1016/j.anndiagpath.2014.09.002 25438927

[8] Fisher C. Synovial sarcoma. Ann Diagn Pathol. (1998) 2:401–21. 10.1016/S1092-9134(98)80042-7 9930576

[9] Spillane AJ A'Hern R Judson IR Fisher C Thomas JM. Synovial sarcoma: a clinicopathologic, staging, and prognostic assessment. J Clin Oncol. (2000) 18:3794–803. 10.1200/JCO.2000.18.22.3794 11078492

[10] Al-Hussaini H Hogg D Blackstein ME O'Sullivan B Catton CN Chung PW. Clinical features, treatment, and outcome in 102 adult and pediatric patients with localized high-grade synovial sarcoma. Sarcoma. (2011) 2011:23. 10.1155/2011/231789 21559258 PMC3087894

[11] Albritton KH Randall RL. Prospects for targeted therapy of synovial sarcoma. J Pediatr Hematol Oncol. (2005) 27:219–2. 10.1097/01.mph.0000163713.46762.72 15838395

[12] Linch M Miah AB Thway K Judson IR Benson C. Systemic treatment of soft-tissue sarcoma-gold standard and novel therapies. Nat Rev Clin Oncol. (2014) 11:187–202. 10.1038/nrclinonc.2014.26 24642677

[13] Wong CS Harris A Kennedy R Houghton OP Carey PD. A rare case of retroperitoneal synovial sarcoma. JRSM Open. (2018) 9:437. 10.1177/2054270418760437 29707226 PMC5912294

[14] Yang J Xu H Guo X Zhang J Ye X Yang Y. Pretreatment inflammatory indexes as prognostic predictors for survival in colorectal cancer patients receiving neoadjuvant chemoradiotherapy. Sci Rep. (2018) 8:3044. 10.1038/s41598-018-21093-7 29445100 PMC5813153

[15] Azab B Shah N Radbel J Tan P Bhatt V Vonfrolio S. Pretreatment neutrophil/lymphocyte ratio is superior to platelet/lymphocyte ratio as a predictor of long-term mortality in breast cancer patients. Med Oncol. (2013) 30:432. 10.1007/s12032-012-0432-4 23283648

[16] Lee S Oh SY Kim SH Lee JH Kim MC Kim KH. Prognostic significance of neutrophil lymphocyte ratio and platelet lymphocyte ratio in advanced gastric cancer patients treated with FOLFOX chemotherapy. BMC Cancer. (2013) 13:350. 10.1186/1471-2407-13-350 23876227 PMC3729814

[17] Feng JF Huang Y Chen QX. Preoperative platelet lymphocyte ratio (PLR) is superior to neutrophil lymphocyte ratio (NLR) as a predictive factor in patients with esophageal squamous cell carcinoma. World J Surg Oncol. (2014) 12:58. 10.1186/1477-7819-12-58 24641770 PMC3973187

[18] Asher V Lee J Innamaa A Bali A. Preoperative platelet lymphocyte ratio as an independent prognostic marker in ovarian cancer. Clin Transl Oncol. (2011) 13:499–503. 10.1007/s12094-011-0687-9 21775277

[19] Siddiqui A Heinzerling J Livingston EH Huerta S. Predictors of early mortality in veteran patients with pancreatic cancer. Am J Surg. (2007) 194:362–6. 10.1016/j.amjsurg.2007.02.007 17693283

[20] Vasquez L León E Beltran B Maza I Oscanoa M Geronimo J. Pretreatment neutrophil-to-lymphocyte ratio and lymphocyte recovery: independent prognostic factors for survival in pediatric sarcomas. J Pediatr Hematol Oncol. (2017) 39:538–46. 10.1097/MPH.0000000000000911 28697168

[21] Kobayashi H Okuma T Oka H Hirai T Ohki T Ikegami M. Neutrophil-to-lymphocyte ratio after pazopanib treatment predicts response in patients with advanced soft-tissue sarcoma. Int J Clin Oncol. (2018) 23:368–74. 10.1007/s10147-017-1199-6 29086877

[22] Xia WK Liu ZL Shen D Lin QF Su J Mao WD. Prognostic performance of pre-treatment NLR and PLR in patients suffering from osteosarcoma. World J Surg Oncol. (2016) 14:127. 10.1186/s12957-016-0889-2 27125872 PMC4850668

[23] García-Ortega DY Álvarez-Cano A Sánchez-Llamas LA Caro-Sanchez CHS Martínez-Said H Luna-Ortiz K. Neutrophil/lymphocyte ratio is associated with survival in synovial sarcoma. Surg Oncol. (2018) 27:551–5. 10.1016/j.suronc.2018.07.012 30217318

[24] Chan JY Zhang Z Chew W Tan GF Lim CL Zhou L. Biological significance and prognostic relevance of peripheral blood neutrophil-to-lymphocyte ratio in soft tissue sarcoma. Sci Rep. (2018) 8:11959. 10.1038/s41598-018-30442-5 30097600 PMC6086886

[25] Nakamura T Matsumine A Matsubara T Asanuma K Uchida A Sudo A. The combined use of the neutrophil-lymphocyte ratio and C-reactive protein level as prognostic predictors in adult patients with soft tissue sarcoma. J Surg Oncol. (2013) 108:481–5. 10.1002/jso.23424 24018883

[26] Szkandera J Gerger A Liegl-Atzwanger B Absenger G Stotz M Samonigg H. Validation of the prognostic relevance of plasma C-reactive protein levels in soft-tissue sarcoma patients. Br J Cancer. (2013) 109:2316–22. 10.1038/bjc.2013.595 24084772 PMC3817333

[27] Gill J Ahluwalia MK Geller D Gorlick R. New targets and approaches in osteosarcoma. Pharmacol Ther. (2013) 137:89–99. 10.1016/j.pharmthera.2012.09.003 22983152

[28] Italiano A Penel N Robin YM Bui B Le CA Piperno-Neumann S. Neo/adjuvant chemotherapy does not improve outcome in resected primary synovial sarcoma: a study of the French Sarcoma Group. Ann Oncol. (2009) 20:425–30. 10.1093/annonc/mdn678 19088169

[29] Palmerini E Staals EL Alberghini M Zanella L Ferrari C Benassi MS. Synovial sarcoma: retrospective analysis of 250 patients treated at a single institution. Cancer. (2009) 115:2988–98. 10.1002/cncr.24370 19452538

[30] Guadagnolo BA Zagars GK Ballo MT Patel SR Lewis VO Pisters PW. Long-term outcomes for synovial sarcoma treated with conservation surgery and radiotherapy. Int J Radiat Oncol Biol Phys. (2007) 69:1173–80. 10.1016/j.ijrobp.2007.04.056 17689031

[31] Wang F Liu ZY Xia YY Zhou C Shen XM Li XL. Changes in neutrophil/lymphocyte and platelet/lymphocyte ratios after chemotherapy correlate with chemotherapy response and prediction of prognosis in patients with unresectable gastric cancer. Oncol Lett. (2015) 10:3411–8. 10.3892/ol.2015.3783 26788143 PMC4665714

[32] Asano Y Kashiwagi S Onoda N Noda S Kawajiri H Takashima T. Predictive value of neutrophil/lymphocyte ratio for efficacy of preoperative chemotherapy in triple-negative breast cancer. Ann Surg Oncol. (2016) 23:1104–10. 10.1245/s10434-015-4934-0 26511266 PMC4773470

[33] Liu T Fang XC Ding Z Sun ZG Sun LM Wang YL. Pre-operative lymphocyte-to-monocyte ratio as a predictor of overall survival in patients suffering from osteosarcoma. FEBS Open Bio. (2015) 5:682–7. 10.1016/j.fob.2015.08.002 26380812 PMC4556728

[34] Chen L Zhang F Sheng XG Zhang SQ. Decreased pretreatment lymphocyte/monocyte ratio is associated with poor prognosis in stage Ib1-IIa cervical cancer patients who undergo radical surgery. Onco Targets Ther. (2015) 8:1355–62. 10.2147/OTT.S82174 26089685 PMC4467643

[35] Keam B Ha H Kim TM Jeon YK Lee SH Kim DW. Neutrophil to lymphocyte ratio improves prognostic prediction of International Prognostic Index for patients with diffuse large B-cell lymphoma treated with rituximab, cyclophosphamide, doxorubicin, vincristine and prednisone. Leuk Lymphoma. (2015) 56:2032–8. 10.3109/10428194.2014.982642 25382617

[36] Gabrilovich DI Ostrand-Rosenberg S Bronte V. Coordinated regulation of myeloid cells by tumors. Nat Rev Immunol. (2012) 12:253–68. 10.1038/nri3175 22437938 PMC3587148

[37] Nozawa H Chiu C Hanahan D. Infiltrating neutrophils mediate the initial angiogenic switch in a mouse model of multistage carcinogenesis. Proc Natl Acad Sci USA. (2006) 103:12493–8. 10.1073/pnas.0601807103 16891410 PMC1531646

[38] Shaw JP Chuang N Yee H Shamamian P. Polymorphonuclear neutrophils promote rFGF-2-induced angiogenesis in vivo. J Surg Res. (2003) 109:37–42. 10.1016/S0022-4804(02)00020-3 12591233

[39] Tazawa H Okada F Kobayashi T Tada M Mori Y Une Y. Infiltration of neutrophils is required for acquisition of metastatic phenotype of benign murine fibrosarcoma cells: implication of inflammation-associated carcinogenesis and tumor progression. Am J Pathol. (2003) 163:2221–32. 10.1016/S0002-9440(10)63580-8 14633597 PMC1892401

[40] Condeelis J Pollard JW. Macrophages: obligate partners for tumor cell migration, invasion, and metastasis. Cell. (2006) 124:263–6. 10.1016/j.cell.2006.01.007 16439202

[41] Liu Y Cao X. The origin and function of tumor-associated macrophages. Cell Mol Immunol. (2015) 12:1–4. 10.1038/cmi.2014.83 25220733 PMC4654376

[42] Dunn GP Old LJ Schreiber RD. The immunobiology of cancer immunosurveillance and immunoediting. Immunity. (2004) 21:137–48. 10.1016/j.immuni.2004.07.017 15308095

[43] Tadmor T Polliack A. Lymphopenia a simple prognostic factor in lymphoma and other cancers: why not use it more as a guide. Leuk Lymphoma. (2010) 51:1773–4. 10.3109/10428194.2010.508825 20849382

[44] Jakóbisiak M Lasek W Gołab J. Natural mechanisms protecting against cancer. Immunol Lett. (2003) 90:103–22. 10.1016/j.imlet.2003.08.005 14687712

[45] Bruckner HW Lavin PT Plaxe SC Storch JA Livstone EM. Absolute granulocyte, lymphocyte, and moncyte counts. Useful determinants of prognosis for patients with metastatic cancer of the stomach. JAMA. (1982) 247:1004–6. 10.1001/jama.247.7.1004 7035703

[46] Elias EG Leuchten JM Buda BS Brown SD. Prognostic value of initial mononucleated cell percentages in patients with epidermoid carcinoma of the head and neck. Am J Surg. (1986) 152:487–90. 10.1016/0002-9610(86)90210-2 3777326

[47] Subimerb C Pinlaor S Lulitanond V Khuntikeo N Okada S McGrath MS. Circulating CD14^+^ CD16^+^ monocyte levels predict tissue invasive character of cholangiocarcinoma. Clin Exp Immunol. (2010) 161:471–9. 10.1111/j.1365-2249.2010.04200.x 20636398 PMC2962964

[48] Petrie HT Klassen LW Kay HD. Inhibition of human cytotoxic T lymphocyte activity in vitro by autologous peripheral blood granulocytes. J Immunol. (1985) 134:230–4. 3871101

[49] Nagaraj S Schrum AG Cho HI Celis E Gabrilovich DI. Mechanism of T cell tolerance induced by myeloid-derived suppressor cells. J Immunol. (2010) 184:3106–16. 10.4049/jimmunol.0902661 20142361 PMC2832724

[50] Kobayashi M Kubo T Komatsu K Fujisaki A Terauchi F Natsui S. Changes in peripheral blood immune cells: their prognostic significance in metastatic renal cell carcinoma patients treated with molecular targeted therapy. Med Oncol. (2013) 30:556. 10.1007/s12032-013-0556-1 23539200

[51] Gasic GJ Gasic TB Stewart CC. Antimetastatic effects associated with platelet reduction. Proc Natl Acad Sci USA. (1968) 61:46–52. 10.1073/pnas.61.1.46 5246932 PMC285903

[52] Nieswandt B Hafner M Echtenacher B Männel DN. Lysis of tumor cells by natural killer cells in mice is impeded by platelets. Cancer Res. (1999) 59:1295–300. 10096562

[53] Bambace NM Holmes CE. The platelet contribution to cancer progression. J Thromb Haemost. (2011) 9:237–49. 10.1111/j.1538-7836.2010.04131.x 21040448

[54] Labelle M Begum S Hynes RO. Direct signaling between platelets and cancer cells induces an epithelial-mesenchymal-like transition and promotes metastasis. Cancer Cell. (2011) 20:576–90. 10.1016/j.ccr.2011.09.009 22094253 PMC3487108

[55] He W Yin C Guo G Jiang C Wang F Qiu H. Initial neutrophil lymphocyte ratio is superior to platelet lymphocyte ratio as an adverse prognostic and predictive factor in metastatic colorectal cancer. Med Oncol. (2013) 30:439. 10.1007/s12032-012-0439-x 23307251

[56] Ohki S Shibata M Gonda K Machida T Shimura T Nakamura I. Circulating myeloid-derived suppressor cells are increased and correlate to immune suppression, inflammation and hypoproteinemia in patients with cancer. Oncol Rep. (2012) 28:453–8. 10.3892/or.2012.1812 22614133

